# Editorial: Long-lasting neurobehavioral effects of early-life events

**DOI:** 10.3389/fnins.2024.1509723

**Published:** 2024-11-19

**Authors:** Rossella Ventura, Matteo Di Segni, Mónica Santos, Carmen Agustín-Pavón, Jose V. Torres-Pérez

**Affiliations:** ^1^Department of Psychology, Sapienza University of Rome, Rome, Italy; ^2^Department of Psychology, Sapienza University, IRCCS San Raffaele, Rome, Italy; ^3^Child Psychopathology Unit, IRCCS Eugenio Medea, Bosisio Parini, Italy; ^4^CNC-Centre for Neuroscience and Cell Biology, University of Coimbra, Coimbra, Portugal; ^5^CIBB-Center for Innovative Biomedicine and Biotechnology, University of Coimbra, Coimbra, Portugal; ^6^Institute for Interdisciplinary Research, University of Coimbra, Coimbra, Portugal; ^7^Department of Cell Biology, Functional Biology and Physical Anthropology, Faculty of Biological Sciences, University of Valencia, Burjassot, Spain

**Keywords:** gene-environment interactions, early-life enrichment, early-life stress, maternal separation, stress-reactivity

Early-life experiences have profound and lasting effects on brain development, physiology, and behavior. These experiences, whether positive or adverse, shape our neural pathways, synaptic communication and metabolic functions in adulthood (Hakamata et al., 2022; Han et al., 2023). Moreover, the way early-life experiences shape final outcomes results from a complex gene-environment interaction that remains not fully understood. The impact of such early-life experiences is not limited to humans, as other animals are also subjected to similar regulation (van Praag et al., 2000; Lyons et al., 2010), which make them valuable models for research in this area. Yet, despite the growing body of literature, the mechanisms by which early-life events interact with genetic predispositions remain elusive. In this Research Topic, we aimed to explore different animal models and selected human populations to dissect these interactions, shedding light on variables often overlooked in this field, including sex, chemical environment, social interactions, and genetic background.

Sasaki et al. explored in mice the effects of perinatal exposure to acephate, an organophosphate insecticide known for its neurodevelopmental toxicity. They demonstrate that early exposure to acephate significantly impacts adult cognitive functions, with sex-linked differences. Male mice showed impairments in short-term memory formation, and reduced neural plasticity markers in the hippocampus, while females exhibited fewer alterations. Importantly, both sexes experienced reduced gut microbiota diversity, suggesting that acephate exposure could lead to long-term alterations in the gut-brain axis. This work highlights the importance of environmental factors in developmental studies, especially as endocrine-disrupting chemicals may differentially affect males and females (Diamanti-Kandarakis et al., 2009; Palanza et al., 2021). This work also highlights the fact that gut microbiota can mediate the cognitive effects of early-life exposure (Sarkar et al., 2021), reinforcing the necessity of integrative approaches to assess neurodevelopment.

Mojahed et al. delved into the neural mechanisms linking early-life traumatic stress and aggression. They found that, in mice, acute social defeat during adolescence results in lasting aggressive behavior, which seems mediated via the posterior ventral medial amygdala (MeApv) and the ventrolateral ventromedial hypothalamus (VmHvl). Interestingly, they also showed how inhibiting excitatory neurons in the MeApv can reduce aggressive tendencies without affecting other social behaviors. By supporting the notion that the amygdala can be a good target to prevent the escalation of aggression, a finding that seems to extend to both sexes (Abellán-Álvaro et al., 2022), this research has significant implications for therapeutics to mitigate aggression in individuals who have experienced early trauma.

In their brief research report, Abellán-Álvaro et al. investigated how maternal separation, a model of early-life chronic stress, affects structural plasticity in the olfactory system of mice. They report an increase in doublecortin-positive cells specifically in the piriform cortex of stressed mice, suggesting that early stress might decrease the maturation and circuit-integration of these embryonically born cells. This neurobiological change was accompanied by increased exploratory behavior in adulthood, while anxiety-like behaviors and corticosterone levels were unaffected. This finding suggests that, instead of inducing pathological anxiety, certain types of early-life stress could be promoting behavioral adaptability. Moreover, Abellán-Álvaro et al. also report a sex-specific increase in body weight in maternally separated males, which highlights the complex interactions between stress, sex and metabolism, and neurodevelopment.

The review article by Matsushima et al. provides a novel perspective on the use of the domestic chick (*Gallus gallus domesticus*) as a model for studying social impairments akin to those seen in autism spectrum disorder (ASD). Chicks, like humans, display early social predispositions (Versace et al., 2017) that, when impaired, can lead to maladaptive socialization patterns (Adiletta et al., 2021). Their review further emphasizes the potential of this animal model for understanding the evolutionary and developmental underpinnings of ASD, particularly in relation to developmental homology, impaired imprinting, and altered social preferences.

The study by Zhang et al. shifts the focus to human health by assessing the predictive value of synthetic MRI in extremely preterm infants. Their study found that specific relaxation times in different brain regions strongly correlate with neurodevelopmental outcomes, particularly in infants with low-grade Germinal Matrix-Intraventricular Hemorrhage (GMH-IVH). The use of synthetic MRI to predict cognitive and motor disabilities demonstrates its potential as a non-invasive tool for early diagnosis. By enhancing predictive accuracy through the combination of multiple MRI metrics, Zhang et al.'s work provides a promising strategy for identifying at-risk infants and thus tailoring early interventions.

Wang et al. utilized Mendelian randomization to explore the causal relationship between early adversity and changes in cortical brain structure. Their findings suggest that early adversity influences specific cortical regions, potentially leading to alterations that underlie various mental disorders. While they could not find a global association, region-specific effects were observed at, among others, the anterior cingulate cortex, which may be relevant to comprehend how childhood adversity contributes to the development of psychopathology (Miu et al., 2022). This study adds a genetic dimension to understanding early-life stress, suggesting that certain individuals may be genetically predisposed to structural brain changes in response to adversity.

Finally, Thao et al. examined the long-term effects of dioxin exposure on white matter microstructure in men from dioxin-contaminated areas in Vietnam. They observed significant alterations in white matter integrity, particularly in those individuals who were exposed perinatally. The reduction in fractional anisotropy in specific brain tracts suggests that dioxin exposure may disrupt neural connectivity, potentially associated with neurodevelopmental and neurodegenerative disorders (Thao et al., 2023). This study, together with Sasaki's research in mice, underscores the long-lasting impact of environmental toxins/chemicals on brain development.

Together, the articles within this Research Topic offer a deeper insight into the pathways linking early interventions to adulthood, highlighting the value of animal models to uncover novel therapeutic strategies to mitigate adverse effects during early-life and to promote resilience in vulnerable populations. From chemical exposures to social stressors and genetic predispositions, these articles provide an ample view of how various early-life factors shape long-term outcomes. As we continue to unravel these intricate interactions, the translational potential of these findings for human health becomes ever more apparent.
